# Glucose or Altered Ceramide Biosynthesis Mediate Oxygen Deprivation Sensitivity Through Novel Pathways Revealed by Transcriptome Analysis in *Caenorhabditis elegans*

**DOI:** 10.1534/g3.116.031583

**Published:** 2016-08-05

**Authors:** Mary L. Ladage, Skylar D. King, David J. Burks, Daniel L. Quan, Anastacia M. Garcia, Rajeev K. Azad, Pamela A. Padilla

**Affiliations:** * Department of Biological Sciences, University of North Texas, Denton, Texas 76203; † Department of Mathematics, University of North Texas, Denton, Texas 76203

**Keywords:** lipid biosynthesis, glucose toxicity, xenobiotic and endobiotic detoxification, innate immunity, collagen

## Abstract

Individuals with type 2 diabetes display metabolic abnormalities, such as hyperglycemia, increased free fatty acids, insulin resistance, and altered ceramide levels, that contribute to vascular dysfunctions and compromised oxygen delivery. *Caenorhabditis elegans* fed a glucose-supplemented diet or with altered ceramide metabolism, due to a *hyl-2* mutation, are sensitive to oxygen deprivation (anoxia). Our experiments showed that the combination of these factors further decreased the anoxia survival. RNA-sequencing analysis was performed to assess how a glucose-supplemented diet and/or a *hyl-2* mutation altered the transcriptome. Comparison analysis of transcripts associated with anoxia-sensitive animals [*hyl-2(tm2031)* mutation or a glucose diet] revealed 199 common transcripts encoded by genes with known or predicted functions involving innate immunity, cuticle function (collagens), or xenobiotic and endobiotic phase I and II detoxification system. Use of RNA interference (RNAi) to target gene products of the xenobiotic and endobiotic phase I and II detoxification system (UDP-glycosyltransferase and Cytochrome p450 genes; *ugt-15*, *ugt-18*, *ugt-19*, *ugt-41*, *ugt-63*, *cyp-13A12*, *cyp-25A1*, and *cyp-33C8*) increased anoxia survival in wild-type animals fed a standard diet. Anoxia sensitivity of the *hyl-2(tm2031)* animals was suppressed by RNAi of *cyp-25A1* or *cyp-33C8* genes. A glucose diet fed to the P0 hermaphrodite decreased the anoxia survival of its F1 embryos; however, the RNAi of *ugt-63* and *cyp-33C8* suppressed anoxia sensitivity. These studies provide evidence that the detoxification system impacts oxygen deprivation responses and that *C. elegans* can be used to model the conserved detoxification system.

Reduced oxygen delivery is central to may human health issues including stroke, cardiac or pulmonary dysfunction, ischemia, and trauma resulting in blood loss or suffocation. Organisms have evolved mechanisms to respond to a lack of oxygen by altering physiological, metabolic, and molecular functions (Hochachka and Lutz 2001; Jonz *et al.* 2016; Nathaniel *et al.* 2015). Despite response and rescue efforts initiated during oxygen deprivation, a continual lack of oxygen can irreversibly damage tissues and cause death. Vascular dysfunction will also reduce blood flow and oxygen delivery, which in turn compromises tissue functions. Individuals with type 2 diabetes display metabolic abnormalities (hyperglycemia, increased free fatty acids, and insulin resistance) that contribute to both microvascular and macrovascular dysfunctions eventually leading to organ dysfunction, tissue damage, blindness, and amputations (Ogden *et al.* 2012; Paneni *et al.* 2013; Beckman *et al.* 2013; Creager *et al.* 2003). There is evidence that individuals with type 2 diabetes have a worse recovery when challenged with an ischemic event. For example, the human diabetic heart is more vulnerable to ischemic injury (Paulson 1997). Additionally, a human clinical study of patients (4537 patients, 937 diabetic patients) hospitalized for a first-time stroke, addressed if stroke subtype, severity, and prognosis differed between diabetic and nondiabetic patients 3 months after stroke (Megherbi *et al.* 2003). Overall, the diabetic patients, compared to nondiabetic patients, were significantly more likely to have motor deficits, limb weaknesses, dysarthria, a higher handicap and disability index, and a significant increase in ischemic stroke. Although studies indicate that type 2 diabetes may worsen the outcome of an ischemic event, it is not yet completely understood why this is the case.

Altered ceramide levels can mediate insulin resistance and mitochondrial dysfunction in diabetics (Xia *et al.* 2014; Novgorodov *et al.* 2016; Turpin *et al.* 2014; Galadari *et al.* 2013). Ceramides, produced by the sphingolipid/ceramide biosynthetic pathway in the endoplasmic reticulum (ER), function within the ER and mitochondria (Hannun and Obeid 2011). This complex set of lipids has roles in various cellular processes including apoptosis, stress, and the mitochondrial unfolded protein response (Deng *et al.* 2008; Liu *et al.* 2014; Hannun and Obeid 2011; Xia *et al.* 2014; Turpin *et al.* 2014; Larsen and Tennagels 2014; Novgorodov and Gudz 2011; Argraves *et al.* 2011; Zigdon *et al.* 2013; Li and Zhang 2013). The finding that specific ceramide species are associated with diseases involving mitochondrial dysfunction has fueled further investigation of these complex molecules (Lopez *et al.* 2013; Larsen and Tennagels 2014; Xia *et al.* 2014). Furthermore, the ability to conduct phenotype analysis of gene mutations impacting the sphingolipid/ceramide pathway is valuable to understanding the functional role of these lipids (Zhang *et al.* 2013; Voelzmann and Bauer 2010). For example, in *Caenorhabditis elegans*, genetic analyses of mutants impacting ceramide/sphingomyelin biosynthesis showed changes in cellular ceramide species (Menuz *et al.* 2009), oxygen deprivation (anoxia) responses (Menuz *et al.* 2009; Garcia *et al.* 2015), the mitochondrial surveillance system (Liu *et al.* 2014), apoptotic signals in the germline (Deng *et al.* 2008), and dietary restriction-induced autophagy and lifespan (Mosbech *et al.* 2013; Cutler *et al.* 2014). Genetic mutations in the ceramide biosynthetic genes alter ceramide species length such that efficient synthesis of C_20_ to C_22_ ceramide and sphingomyelin species requires *hyl-2* and efficient synthesis of C_24_ and C_26_ ceramide and sphingomyelin requires *hyl-1* (Menuz *et al.* 2009). A mutation in *hyl-2* results in oxygen deprivation sensitivity and suppresses the anoxia resistance observed in the *daf-2(e1370)* insulin receptor mutant (Menuz *et al.* 2009; Garcia *et al.* 2015). Conversely, a mutation in *hyl-1* moderately increases oxygen deprivation survival (Menuz *et al.* 2009). The mechanism linking ceramide species and anoxia survival remains to be determined.

*C. elegans* is a well-established model for studying oxygen deprivation at the molecular level since various stages of development are capable of surviving a broad range of oxygen levels (Van Voorhies and Ward 2000; Padilla *et al.* 2002). In fact, the worm is capable of surviving severe oxygen deprivation (anoxia) by entering into a hypometabolic state, referred to as suspended animation, in which observable biological processes (cell division, development, and movement) reversibly arrest (Padilla *et al.* 2002; Padilla and Ladage 2012). Several labs have identified genetic mutations affecting various biological processes (*e.g.*, insulin signaling, ovulation, HIF-1 signaling, and metabolism), that either increase or decrease the oxygen deprivation survival rate (Scott *et al.* 2002; Mendenhall *et al.* 2009, 2006; Jiang *et al.* 2001; Anderson *et al.* 2009). The *C. elegans* nervous system can sense small changes in oxygen levels (Zimmer *et al.* 2009; Gray *et al.* 2004) and methodologies have been developed to use *C. elegans* as a model for hypoxic injury and ischemia/reperfusion (Queliconi *et al.* 2014; Fawcett *et al.* 2015; Mao *et al.* 2016). Interestingly, a sugar-supplemented diet reduces oxygen deprivation survival and feeding the wild-type worms the diabetic drug metformin enhances anoxia survival (LaRue and Padilla 2011; Garcia *et al.* 2015). A glucose-supplemented diet can also be considered an obesity mimetic as it increases lipid accumulation as seen by oil red O staining (Garcia *et al.* 2015). Furthermore, a glucose diet decreases membrane fluidity in worms with altered fatty acid metabolism (*paqr-2* and *iglr-2* mutants), alters gene expression, impacts lifespan, and increases cellular ROS levels and protein glycosylation (Svensk *et al.* 2016; Mondoux *et al.* 2011; Choi 2011; Schlotterer *et al.* 2009; Lee *et al.* 2009; Garcia *et al.* 2015; Liggett *et al.* 2015). Dietary glucose impacts various physiological and molecular processes in *C. elegans*, which makes it a good model for understanding hyperglycemia and obesity.

In this study, we begin to address why a glucose diet or a mutation in the *hyl-2* ceramide synthase gene reduces anoxia survival. The *hyl-2(tm2031*) mutant, compared to wild-type controls, is more sensitive to anoxia when fed a glucose diet. RNA-sequencing (RNA-Seq) analysis indicates the *hyl-2(tm2031)* and N2 animals fed a glucose diet have overlapping but not identical gene expression responses and that the transcript levels of lipid metabolic genes are altered. Comparison analysis of the transcriptome profiles associated with anoxia sensitive animals (*hyl-2(tm2031)* mutation, wild-type fed a glucose diet) revealed altered expression levels of 199 common transcripts involved with various functions including innate immunity, cuticle function, and the xenobiotic and endobiotic phase I and II detoxification system (referred to here as the detoxification system). The detoxification system is understudied in *C. elegans*, but has been well characterized in humans, especially in the context of drug metabolism. These enzymes regulate the levels of potentially toxic lipophilic molecules through oxidation and sugar conjugation, which facilitates elimination by decreasing the hydrophobicity of the target molecule. RNAi knockdown of *cyp* (cytochrome P450, phase I) and *ugt* (UDP-glucuronlytransferases, phase II) detoxification genes increased anoxia survival and in some cases suppressed anoxia sensitivity induced by the *hyl*-2 mutation or a glucose diet. These are the first studies that provide evidence that changes in the detoxification system suppress oxygen deprivation sensitivity induced by a glucose diet or altered ceramide species.

## Materials and Methods

### Strains and culture conditions

The wild-type Bristol (N2) and *hyl-2(tm2031)* strains were cultured using NGM plates seeded with *Escherichia coli* (OP50 or HT115) and raised at 20°. To obtain animals of the specified stage of development for each experiment as noted below, 1 d old adult hermaphrodites were allowed to lay eggs on a plate (1–2 hr), and anatomical markers such as gonad morphology and time post molting stage were used to identify the developmental stage of the subsequent offspring. Animals were fed a glucose-supplemented diet as described previously (Garcia *et al.* 2015). Glucose (Sigma) solution was spread evenly, fully covering the entire plate, and allowed to dry before being seeded with bacteria (OP50, HT115, or appropriate RNAi strain). To minimize differences between the bacterial lawns, freshly made plates were used within a 7 d period. To examine how a glucose diet impacts the F1 generation, hermaphrodites were raised on the glucose diet to adulthood, placed onto a fresh NGM glucose plate, allowed to lay eggs for approximately 2 hr and removed, so that the F1 generation of embryos could be phenotypically examined.

### RNAi analysis

RNA interference (RNAi) was conducted as described (Kamath and Ahringer 2003; LaRue and Padilla 2011). The HT115 and RNAi *E. coli* strains were obtained from the MRC Geneservice or Source BioSciences (Cambridge, UK). Embryos were placed on to NGM-IPTG plates (200 mg/ml ampicillin, 12.5 mg/ml tetracycline, and 0.5 mg/ml IPTG) seeded with or without glucose and either HT115 control food (contains L4440 plasmid with no insert) or the appropriate *E. coli* strain for RNAi of a specified gene. Embryos were grown to 1 d old adults at 20° and then exposed to anoxia as described below.

### Anoxia experiments

Animals were placed into anoxia as previously described (Padilla *et al.* 2002, 2011). Briefly, animals at the indicated developmental state were exposed to anoxia (20°) for the time indicated for each experiment (1 or 2 d of exposure) using the BD Biobag type A anaerobic environmental chamber (BD Biosciences, Rockville, MD). Animals were given 24 hr of recovery in air (20°) before survivors were examined for an unimpaired or impaired phenotype, as previously described (LaRue and Padilla 2011; Garcia *et al.* 2015). The survivors were scored as unimpaired if there were no detectable defects in morphology, behavior, or motility using a standard stereomicroscope, whereas impaired animals displayed a defect in morphology, behavior, or motility. A minimum of three independent experiments of approximately 50 animals each was conducted. Statistical analysis of total survival rate relative to control was conducted as indicated for each experiment. Tests conducted include one-way ANOVA, Bonferonni or Tukey multiple comparisons or two-way ANOVA, and Sidak’s multiple comparisons test if genotype and developmental stage or genotype and glucose concentration were analyzed (Prism 6.0).

### Fecundity assays

The number of progeny, produced by *hyl-2(tm2031)* or N2 animals, was determined as previously described (Mendenhall *et al.* 2009). Briefly, four synchronized animals were collected at the L4-to-adult molt, placed as individuals onto a NGM plate, and allowed to lay eggs over a 24 hr period. The adult worms were moved every 24 hr and the progenies produced during each 24 hr interval were counted after hatching. Animals were examined until no progenies were produced. For statistical analysis, a two-way ANOVA, Sidak’s multiple comparisons test was conducted relative to genotype and adult stage. The total numbers of offspring, for N2 and *hyl-2(tm2031)* animals, was statistically analyzed using an unpaired *t*-test (two-tailed). For all statistical analyses Prism 6.0 was used.

### RNA extraction and sequencing

Young nongravid adults were collected for mRNA isolation as previously described (Garcia *et al.* 2015). RNA sampling was done in three biological replicates for *hyl-2(tm2031)* animals fed either OP50 or glucose-supplemented OP50 diet (0.5% glucose). Briefly, gravid adults were allowed to lay eggs on several NGM plates for 2–4 hr, adults were then washed off with M9, and the embryos, which remained on the plate, were allowed to hatch. L1 animals were collected and placed on respective media plates (OP50 only or OP50 supplemented with 0.5% glucose). Approximately 10,000 animals (per trial) were grown to young adulthood, past the L4 molt but prior to egg production, before collection by gravity separation with M9 in 15 ml conical tubes. The animals were washed 3 times with M9 and allowed to settle by gravity separation to remove bacteria. Excess M9 was removed, and 4 vol of Trizol per vol of animals were added. The tubes were immediately frozen in liquid nitrogen, thawed and briefly vortexed; this step was repeated and then followed by a 5 min incubation at room temperature. To each tube, 0.2 ml of chloroform per 1 ml of Trizol was added, mixed gently for 15 sec, incubated at room temperature for 2–3 min, and then centrifuged at 12,000 × *g* for 15 min at 4°. The colorless upper phase was transferred to an RNase free tube. RNA was then purified using an Ambion PureLink RNA Mini Kit (Cat# 12183018A). Following RNA purification, the RNA was treated with DNase I (Life Technologies, Cat# 12185010), quantified using a NanoDrop Spectrophotometer and stored at −80°. RNA was isolated from three independent experiments. Next-generation RNA sequencing (RNA-Seq) experiments and analysis was done as previously described (Garcia *et al.* 2015). The RNA-Seq was done at the University of Texas Southwestern Genomics Core facility, which resulted in generation of approximately 30.86 M, 35.42 M, and 30.78 M single-end reads of length 50 bp for the three *hyl-2(tm2031)* OP50-fed replicates (mean 32.35 million reads), and 30.31 M, 29.79 M, and 31.56 M single-end reads of length 50 bp for the three *hyl-2(tm2031)* glucose-fed replicates (mean 30.56 million reads). These deep sequencing raw transcriptome data were then used for a comprehensive quantitative analysis of differential gene regulation in *hyl-2(tm2031)* and glucose-fed *C. elegans*. The publicly available Tuxedo suite of programs was used for RNA-Seq analysis, which includes the software Bowtie (Langmead *et al.* 2009), Tophat (Trapnell *et al.* 2009), and Cufflinks (Trapnell *et al.* 2010). The *C. elegans* genome build WS195 at NCBI was used as the reference genome for obtaining the read alignment using Bowtie and the splice junctions using Tophat. Cufflinks was used to identify the genes in glucose-fed *C. elegans* with significant alteration in their expressions compared to wild-type *C. elegans*. Cufflinks performs a statistical significance test for differential expression of each transcript based on a negative binomial model estimated from the data (Trapnell *et al.* 2010). The transcripts passing the significance test [False discovery rate (FDR) adjusted *P*-value to be < 0.05, Trapnell *et al.* 2010] were examined further for their abundance fold change between the conditions. Custom programs were developed for further parsing of this data and classifying the differentially expressed genes based on functional annotation at Wormbase database (http://www.wormbase.org/).

### Gene ontology (GO) and enrichment of functional gene classes

Genes differentially regulated by a glucose diet and/or a *hyl-2(tm2031)* mutation were classified and evaluated using PANTHER (http://www.pantherdb.org) and Wormbase annotation (http://www.wormbase.org) (Mi *et al.* 2013b; Chen *et al.* 2005). The PANTHER classification system combines gene function, ontology, and pathways to analyze large data sets (Mi *et al.* 2013a). The transcripts involved with lipid metabolism or the 199 transcripts common to the anoxia sensitive animals (*hyl-2(tm2031)* and N2 glucose-fed) were analyzed using the web-based Babelomics 5 platform (Al-Shahrour *et al.* 2005; Alonso *et al.* 2015). Single enrichment analysis using Fatigo was performed with the entire *C. elegans* genome as the comparative background (Al-Shahrour *et al.* 2007). GO biological processes, molecular function, and cellular component databases were simultaneously queried. For gene set enrichment analysis, a logistic model method available within the Fatiscan tool was utilized (Al-Shahrour *et al.* 2007). Genes were ranked for analysis using their fragments per kb of transcript per million mapped reads (FPKM) expression values for both the *hyl-2(tm2031)* and N2 supplemented with glucose RNA-Seq experimental datasets.

### Network analysis, clustering, and heatmap generation

To generate the functional-association dataset the 199 transcripts, common to the anoxia sensitive animals, were processed through the GeneMANIA web interface (Warde-Farley *et al.* 2010; Mostafavi *et al.* 2008). The 199 differentially expressed genes were input as query genes to GeneMANIA, which queried against the *C. elegans* organism database. Network weighting was based on biological processes as in GO. Edge weights are automatically determined by GENEMANIA based on the input gene list, and vary by association type. Association types include gene coexpression, protein–protein interaction, shared domains, shared pathways, and colocalization. The parameters were set such that only the 199 differentially expressed genes were analyzed for network interactions and thus no predicted genes outside of this data set were included. To identify gene clusters, the generated network of weighted edges and nodes was downloaded in text format directly from GeneMANIA and converted into the SIF file format using custom awk commands (Aho *et al.* 1987). A protein–protein interaction network for the 199 differentially expressed genes was generated using yeast two-hybrid data available at the Worm Interactome Database (Simonis *et al.* 2009). The interaction dataset thus obtained was converted to SIF file format using custom awk commands for use with the Cytoscape program (Version 3.3.), which allows the visualization and interpretation of interactions (Shannon *et al.* 2003; Simonis *et al.* 2009; Montojo *et al.* 2014). Here, the genes were represented as nodes and the positive yeast two-hybrid interactions as edges between interacting nodes. All networks were further analyzed for functional modules using the Markov Clustering Algorithm (Enright *et al.* 2002). Custom awk commands were used to convert the network data to “abc” format specified in the MCL algorithm manual. The inflation parameter was tested at values ranging from 1 to 6 in order to identify the optimal granularity for clustering (Mao *et al.* 2009). For the GeneMANIA network, an inflation parameter of value 2 was found to produce the clusters for further analysis. Heatmaps, for the 199 common transcripts list and for individual network clusters, were generated with unsupervised hierarchical clustering using the “aheatmap” function of the NMF R library (Gaujoux and Seoighe 2010). The heatmap settings were set such that the differentially expressed fold change value determined the transcript order. Hierarchical clustering was performed using Euclidian distance and complete linkage analysis (Gaujoux and Seoighe 2010).

### Quantitative RT-PCR

Animals were collected as young adults for mRNA isolation and RNA extraction was performed as described above. Reverse transcription to generate cDNA was performed using SuperScript III first-strand synthesis system for RT-PCR (Invitrogen, Cat# 18080-051). Quantitative RT-PCR was carried out using a CFX384 Touch Real-Time PCR Detection System (Bio-Rad) and SsoFast EvaGreen Supermix (Bio-Rad). Following primer validation, results were analyzed using the 2^−ΔΔCT^ Method (Livak and Schmittgen 2001). The mRNA level of Y45F10D.4 was used for normalization (Hoogewijs *et al.* 2008). The average of at least three technical replicates was used for each independent experiment. Primer sequences are available upon request. Statistical analysis was conducted using one-way ANOVA, Bonferroni multiple comparisons.

### Data availability

The data have been archived in NCBI GEO under the series record number GSE83887.

## Results

### Phenotypes associated with the hyl-2(tm2031) animal

**Figure 1:**
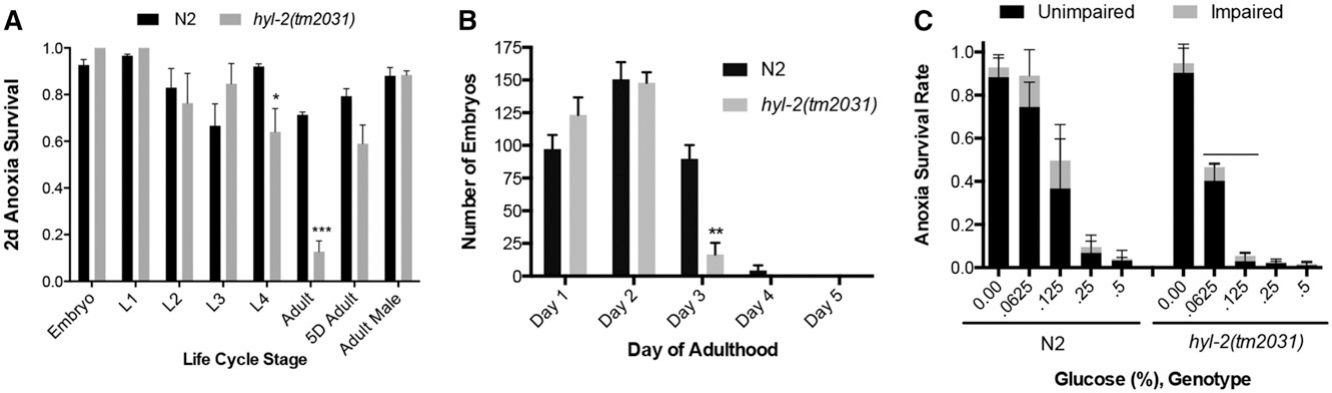
Phenotype of the *hyl-2(tm2031)* mutant. (A) Wild-type and *hyl-2(tm2031)* animals, at larval stages, 1 d or 5 d old adult hermaphrodites, and 1 d old males, were exposed to 2 d of anoxia. The *hyl-2(tm2031)* L4 larvae and adult animals had a significant decrease in anoxia survival relative to N2 controls of the same developmental stage (* indicates *P* < 0.05, *** indicates *P* < 0.0001, two-way ANOVA, Sidak’s multiple comparisons test). (B) The average number of progeny produced in 1–5 d old *hyl-2(tm2031)* adults relative to N2 controls of the same developmental stage. There was a significant difference in the number of progeny produced in 3 d old *hyl-2(tm2031)* adults (** indicates *P* < 0.0001, two-way ANOVA, Sidak’s multiple comparisons test). There was a significant decrease in the total number of offspring produced per *hyl-2(tm2031)* hermaphrodite relative to N2 controls (N2 = 341.8 ± 15.8, *hyl-2(tm2031)* = 287.8 ± 6.3, *P* < 0.05, two-tailed unpaired *t*-test). (C) The *hyl-2(tm2031)* 1 d old adult hermaphrodite is more sensitive to one day of anoxia, after being fed a diet supplemented with glucose prior to anoxia exposure, relative to N2 control animals. Bar indicates a significant decrease in the number of *hyl-2(tm2031)* animals alive in comparison to N2 animals fed the same concentrations of glucose (0.0625%, 0.125%) prior to anoxia exposure (two-way ANOVA, Sidak’s multiple comparisons test). ANOVA, analysis of variance.

To examine the role that ceramides have in anoxia survival, phenotypic analysis and transcriptomic analysis of the *hyl-2(tm2031)* mutant were conducted. Consistent with what others have observed, the *hyl-2(tm2031)* 1 d old adult hermaphrodite exposed to 2 d of anoxia had a decreased survival rate (Menuz *et al.* 2009) (Figure 1A). We also examined the anoxia survival rate of the *hyl-2(tm2031)* animal at other developmental stages and in the adult male, and found that hermaphrodites at the L4 larvae stage were also sensitive to anoxia (Figure 1A). This suggests that a physiological attribute in L4 and 1 d old adult hermaphrodites in combination with altered ceramide metabolism compromises anoxia survival. We noticed that the *hyl-2(tm2031)* mutant also displayed a decrease in average brood size relative to wild-type animals (287.8 ± 6.3 compared to 341.8 ± 15.8, *P* < 0.05). However, the reduced offspring observed in *hyl-2(tm2031)* hermaphrodites is due to a decrease in egg laying in 3 d old adults (Figure 1B). Thus, it is probably not egg production *per se* that is contributing to the anoxia sensitivity observed in 1 d old adult hermaphrodites. Previously, we showed that wild-type animals fed a glucose-supplemented diet have a reduced 1 d anoxia survival rate (Garcia *et al.* 2015). Here we found that the *hyl-2(tm2031)* adult hermaphrodite, fed a low concentration of glucose, has a reduced 1 d anoxia survival rate in comparison to the wild type fed the same glucose diet (Figure 1C). Note that the *hyl-2(tm2031)* mutant fed a standard diet will survive 1 d, but not 2 d, of anoxia exposure. These results indicate that both altered ceramide levels and a glucose diet have an additive and negative impact on anoxia survival and that a glucose diet, more so than the *hyl-2* mutation, reduces the survival of 1 d of anoxia.

### Transcriptional changes induced by an altered ceramide metabolism and a glucose diet

Previously, we reported the transcript profile of wild-type animals fed a glucose-supplemented diet relative to those fed an *E. coli* OP50-only diet and determined that the expression of 2370 genes were altered in response to the glucose-supplemented diet (Garcia *et al.* 2015). Given that ceramide changes (*hyl-2* mutant) and a glucose-supplemented diet negatively impact anoxia survival, we hypothesized that there is an overlap in the transcriptional profile between these two anoxia sensitive animals (*hyl-2(tm2031)* and N2 glucose-fed animals). Furthermore, since the *hyl-2(tm2031)* mutant, relative to N2, is more sensitive to anoxia after being fed a glucose-supplemented diet, we hypothesized that the transcriptome profile of N2 animals fed a glucose diet and that of *hyl-2(tm2031)* animals fed a glucose diet are overlapping but not identical. To test these hypotheses, we used RNA-Seq to identify transcriptional changes in the *hyl-2* mutant fed a standard and a glucose-supplemented diet (0.5%) and compared these datasets to our RNA-Seq results of N2 animals fed a standard diet and those fed a glucose-supplemented diet (Garcia *et al.* 2015). Note that the previously reported transcriptome profiles for the N2 animals and the *hyl-2(tm2031)* transcriptome profiles reported here were conducted at the same time (Garcia *et al.* 2015).

**Figure 2:**
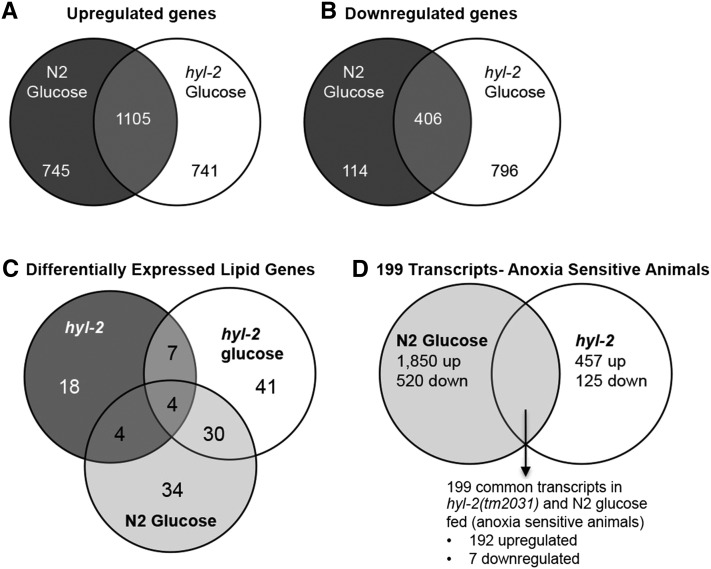
Venn diagram depicts the number of (A) upregulated transcripts or (B) downregulated transcripts identified between N2 and *hyl-2(tm2031)* animals fed a glucose diet. (C) Venn diagram depicts the transcripts that are differentially expressed and predicted to produce lipid metabolism proteins. (D) Venn diagram depicts that 199 transcripts are common between the anoxia sensitive animals (N2 glucose-fed and *hyl-2(tm2031)* animals).

The *hyl-2(tm2031)* animals had 457 genes that were significantly upregulated and 125 genes that were significantly downregulated in comparison to the N2 animals (Supplemental Material, Figure S1A and Table S1). The *hyl-2(tm2031)* animals fed a glucose-supplemented diet had 1846 genes that were significantly upregulated and 1202 genes that were significantly downregulated in comparison to the *hyl-2(tm2031)* animals fed an OP50 only diet (Figure S1B and Table S2). Based on the PANTHER biological processes classification (http://www.pantherdb.org), genes involved with metabolic or cellular processes were the gene classes that displayed the highest number of gene expression changes in the *hyl-2* mutant (Figure S2). We used the Gene List Venn Diagram program (http://genevenn.sourceforge.net) to assess the overlap between the RNA-Seq results of *hyl-2(tm2031)* animals fed a glucose diet to that of N2 wild-type animals fed a glucose diet. The genes that were differentially regulated in response to a glucose diet (upregulated, Figure 2A and Table S3; downregulated, Figure 2B and Table S4) were overlapping but not identical between N2 and *hyl-2(tm2031)* animals. Together, these data indicate that the *hyl-2(tm2031)* mutation impacts gene expression profiles and that the *hyl-2(tm2031)* and N2 animals fed a glucose-supplemented diet do not have identical gene expression responses.

Previously, we determined that, relative to wild-type controls, wild-type animals fed a glucose-supplemented diet have an increase in lipid droplets and *hyl-2(tm2031)* animals fed a standard OP50 diet have a reduction in lipid droplets (as detected by Oil Red O staining) (Garcia *et al.* 2015). Thus, it is not simply that a general increase or decrease of lipid levels results in anoxia sensitivity, and perhaps the anoxia sensitivity is tied to alteration of specific lipid pathways. There are 471 known or predicted genes involved with lipid metabolism in the *C. elegans* genome (Zhang *et al.* 2013). We used the Gene List Venn Diagram program (http://genevenn.sourceforge.net) to identify and compare the lipid metabolism genes that were differentially regulated in *hyl-2(tm2031)* animals, glucose-fed *hyl-2(tm2031)* animals, and glucose-fed wild-type animals (Figure 2C, Table S1, and Table S2). The *hyl-2* mutation and a glucose diet alter the expression profile of lipid genes in an overlapping but not identical manner. Four lipid metabolism genes (*cyp-25A1*, *cyp-13A12*, *cyp-33C8*, and *acs-2*) were differentially expressed, relative to N2 controls fed a standard diet, in the *hyl-2(tm2031)* animals fed a standard or glucose diet, and glucose-fed wild-type animals (Figure 2C and Table S5). We used the Babelomics 5 web interface (babelomics.bioinfo.cipf.es) to conduct an enrichment analysis to find GO terms that were overrepresented in the lipid metabolism gene datasets (Table S5). The GO terms, namely, membrane lipid metabolism, sphingolipid metabolic process, and cellular lipid metabolic process were overrepresented in the *hyl-2(tm2031)*, *hyl-2(tm2031)* fed glucose, and N2 fed glucose animals (Table S5). Genes within these GO term classes include *asm-2*, *cgt-1*, *gba-4*, and *fat-5* (Table S5).

### The transcriptome of anoxia sensitive animals

**Figure 3:**
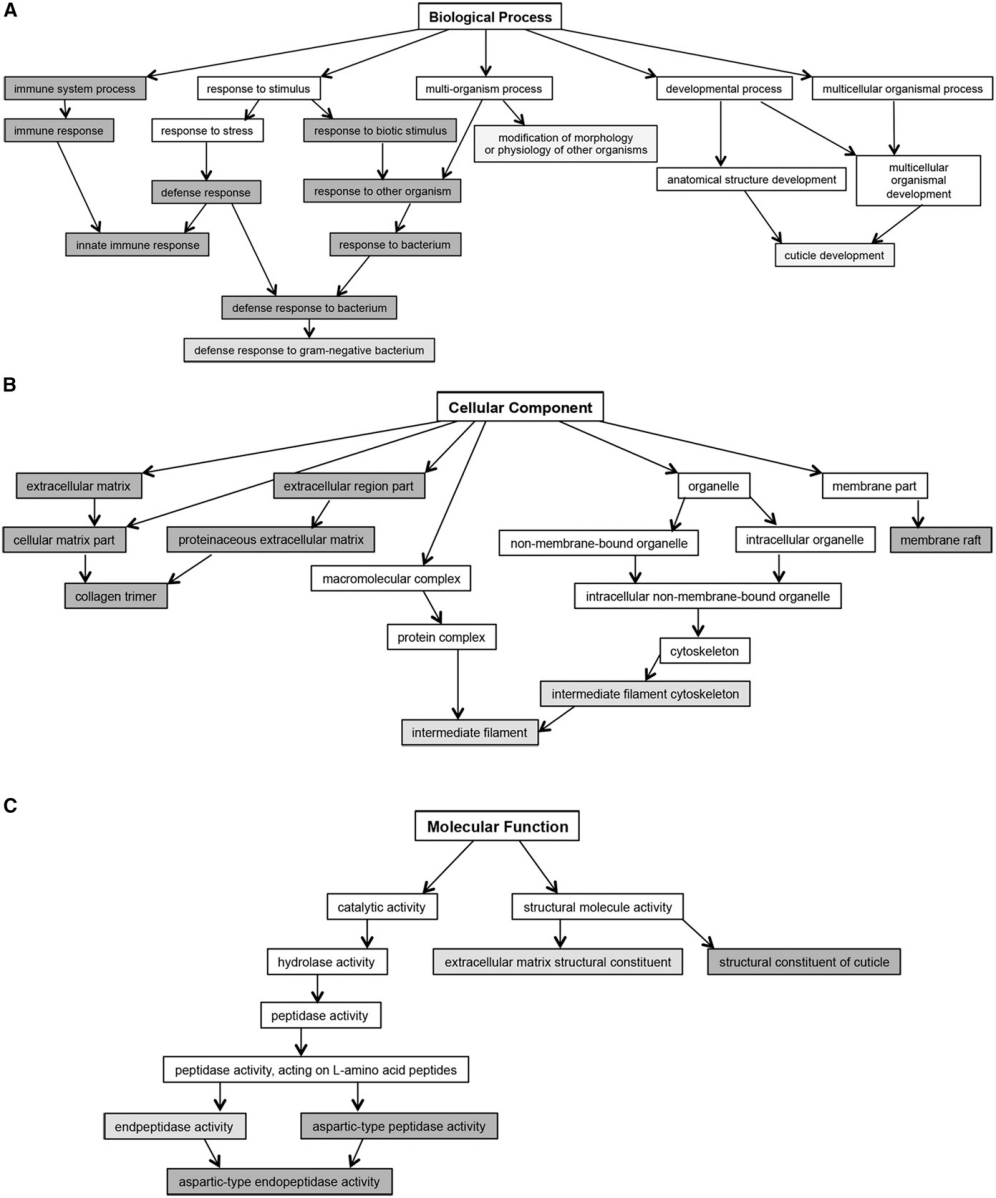
Babelomics 5 was used to identify the predominant significantly enriched GO terms associated with specific (A) biological processes, (B) cellular components, or (C) molecular functions, identified by FatiGO and FatiScan enrichment analysis for the transcriptional profiles of the 199 genes associated with anoxia sensitivity in the N2 animals fed a glucose diet and in the *hyl-2(tm2031)* mutant. Significantly enriched GO terms are represented by shaded nodes with the intensity of shading increasing as adjusted *P*-values decrease below 0.05 using the FDR procedure of Benjamini and Hochberg (1995). FDR, false discovery rate.

Sensitivity to anoxia is observed in wild-type animals fed a glucose diet and in *hyl-2* mutants, indicating that both diet and genotype impact oxygen deprivation responses to varying degrees. To identify transcripts associated with anoxia sensitivity, we compared the RNA-Seq data for the anoxia sensitive animals (N2 glucose-fed, *hyl-2(tm2031)* OP50 diet) relative to wild-type N2 control animals fed a standard OP50 diet (Figure 2D). This revealed 199 common transcripts in the anoxia sensitive animals; 192 transcripts were upregulated and 7 transcripts were downregulated (Figure 2D and Table S6). To identify GO terms that were overrepresented in the 199 common transcripts the Babelomics 5 web interface was used (babelomics.bioinfo.cipf.es). The classification of transcripts by biological processes identified GO terms involved with bacterial defense, innate immunity, and cuticle development (Figure 3A and Table S7); cell component included GO terms associated with the extracellular matrix (ECM), intermediate filament, and membrane raft (Figure 3B and Table S7) and; molecular cell function identified GO terms associated with peptidase activity and the structural constituent of the cuticle (Figure 3C and Table S7).

**Figure 4:**
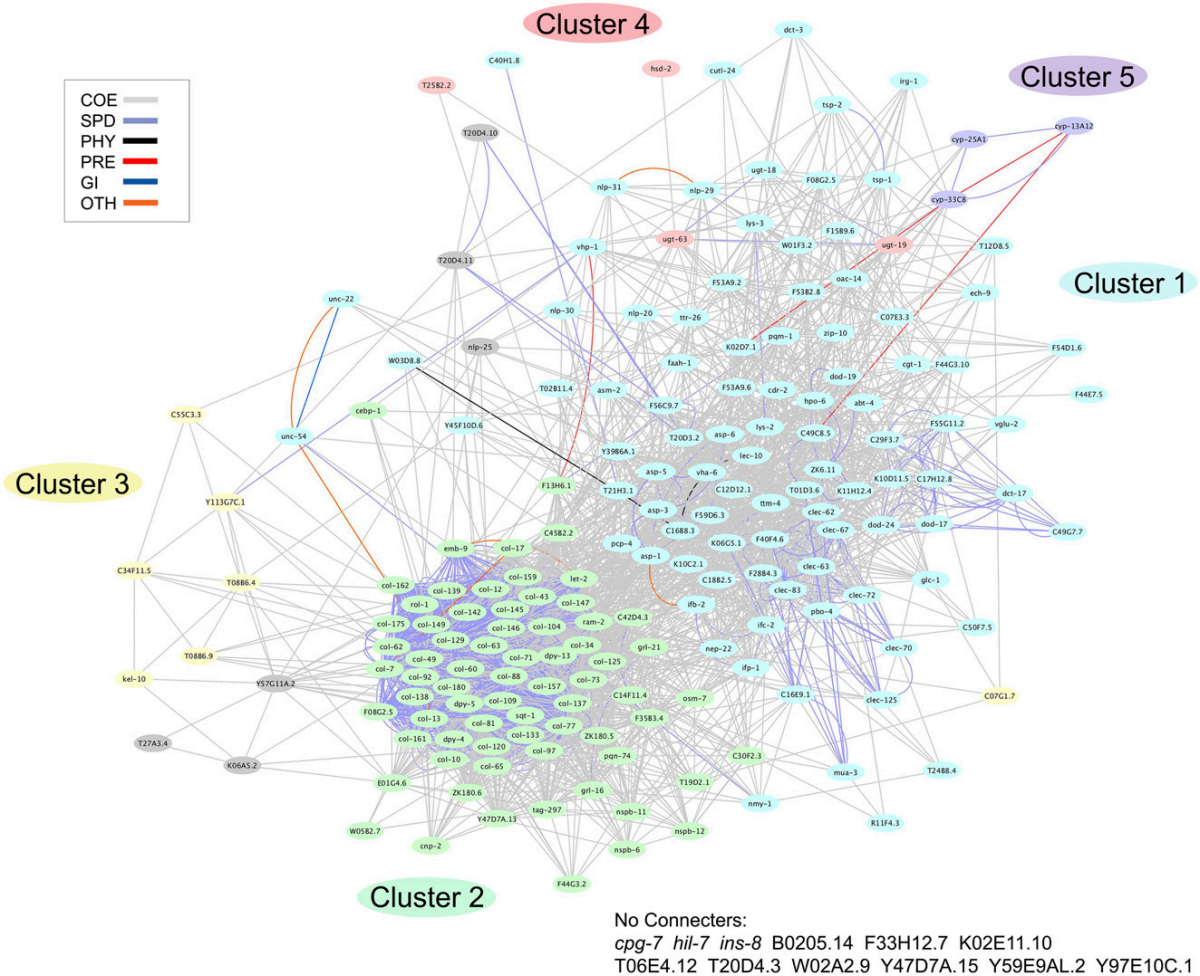
The network for the 199 transcripts associated with anoxia sensitivity. This network of 199 transcripts was analyzed by GeneMANIA to identify genes that are: coexpressed (COE), have similar protein domains (SPD), physically interact (PHY), predicted to interact (PRE), genetically interact (GI), or interact in some other manner (OTH). Further bioinformatics analysis identified two large gene clusters (cluster 1 and 2) and three smaller gene clusters (cluster 3, cluster 4, and cluster 5) within the network.

Visualization of the transcript data, using a heatmap, indicates that many of the 199 common transcripts were upregulated in a similar manner in the anoxia sensitive animals (Figure S3). However, there were some transcripts that were at higher levels in the glucose-fed wild-type animals (Figure S3). The transcripts that were downregulated were at similar levels in the anoxia sensitive animals (Figure S3). The GeneMANIA web interface (http://www.genemania.org) was used to generate a composite functional association network of the 199 genes, which was then analyzed for modularity using a Markov Clustering Algorithm (Enright *et al.* 2002). The composite network consists of various individual functional association networks derived from publicly available genomic and proteomic datasets by GeneMANIA. Weighted connective edges link the 199 genes, referred to as the nodes of the network, based on the Pearson correlation of each gene pair’s corresponding source data. All but 12 of the 199 genes identified were part of an interacting network, which included two large gene clusters (cluster 1 and 2) and three smaller gene clusters (cluster 3–5) (Figure 4 and Table S8). Cluster 1, with 86 genes, includes genes known to be involved in innate immunity, while Cluster 2, with 71 genes, includes genes that code for collagens or are involved in cuticle formation. Cluster 4 and Cluster 5 include genes encoding members of the xenobiotic and endobiotic phase I and II detoxification system (Table S8). Gene expression heatmaps were generated for genes in Cluster 1 (Figure S3B), Cluster 2 (Figure S3C), and combined for Cluster 3, 4, and 5 (Figure S3D). Cluster 1 contains a few genes with higher expression in the *hyl-2(tm2031)* animal relative to the glucose-fed wild-type animal (Figure S3B). Cluster 2 contains many genes displaying higher expression in the glucose-fed wild-type animal relative to the *hyl-2(tm2031)* animal (Figure S3C). Since the Clusters 3, 4, and 5 were smaller, we combined the transcript data into a single gene expression heatmap; these clusters contain genes that were upregulated or downregulated in a similar manner (Figure S3D). Taken together, the use of two independent methods for transcript analysis (GeneMANIA and Babelomics 5) indicates that immune responses and collagen functions could be altered in animals that are sensitive to anoxia exposure.

### Xenobiotic and endobiotic phase I and phase II detoxification system

**Figure 5:**
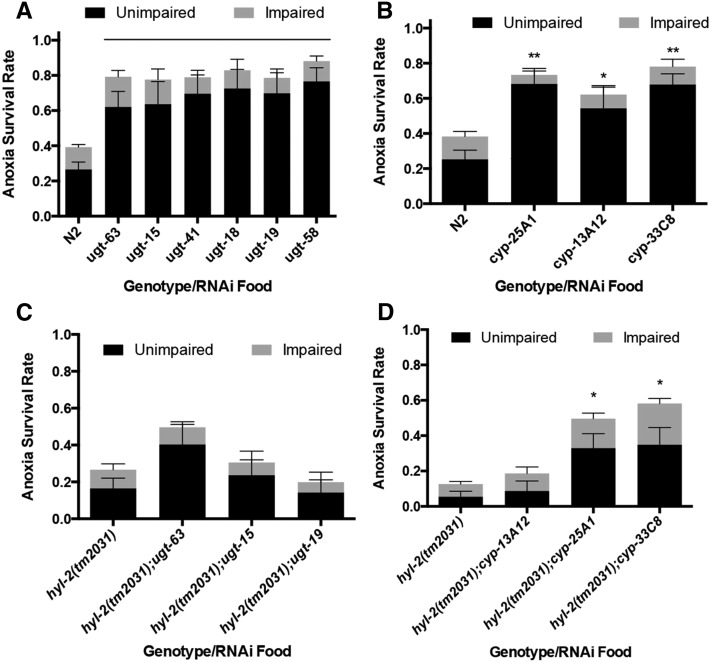
Knockdown of genes predicted to be involved with the xenobiotic and endobiotic phase I and phase II system increases anoxia survival. Relative to N2 wild-type controls, RNAi of *ugt* (A) and *cyp* (B) genes increases survival in 1 d old adult animals exposed to 2 d of anoxia (line and ** indicates *P* < 0.001, * indicates *P* < 0.05). Relative to *hyl-2(tm2031)* control, RNAi of *ugt* did not significantly increase anoxia survival (C), but RNAi of *cyp-25A1* and *cyp-33C8* (D) did increase anoxia survival (* indicates *P* < 0.05). For (A) and (B), animals were raised on the RNAi food or HT115 *E. coli*. with no added glucose. For all, statistical analysis was done using one-way ANOVA, Bonferonni multiple comparisons test; at least three independent experiments, with *n* ≥ 100. ANOVA, analysis of variance; RNAi, RNA interference.

To determine if some of the transcriptional changes revealed by our RNA-Seq analysis alter anoxia responses, we used RNAi to target some genes differentially expressed in the anoxia sensitive animals. First, we examined the phenotype associated with RNAi knockdown of the seven genes downregulated in the anoxia sensitive animals (Table S6). RNAi knockdown of *ugt-63* and *cyp-25A1* resulted in a significant increase in anoxia survival relative to N2 animals, (Figure 5, A and B) but RNAi of the other five genes did not result in a significant difference in anoxia survival (Figure S4). The *ins-8(RNAi)* and *F44E7.5(RNAi)* animals exposed to anoxia had an inconsistent and variable anoxia survival rate; additional analysis would be needed to determine if changes in these genes impact the anoxia response (Figure S4).

**Figure 6:**
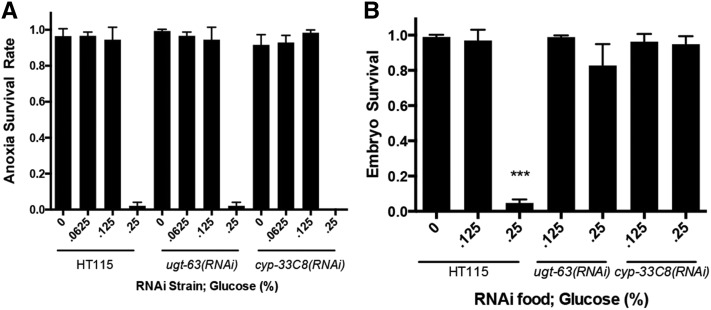
Knockdown of genes predicted to be involved with the xenobiotic and endobiotic phase I and phase II system increases anoxia survival in the offspring of hermaphrodites fed a glucose diet. (A) Relative to N2 wild-type controls, RNAi of *ugt-63* or *cyp-33C8* did not increase anoxia survival in 1 d old adult animals fed a glucose diet. (B) Wild-type adult animals fed a glucose diet (0.25%) produce embryos that are unable to survive anoxia exposure. RNAi of *ugt-63* or *cyp-33C8* suppressed the anoxia sensitivity observed in embryos from adults fed a glucose diet (*** indicates *P* < 0.0001 using one-way ANOVA, Tukey multiple comparisons test; at least three independent experiments, with *n* > 50 embryos per experiment). ANOVA, analysis of variance; RNAi, RNA interference.

Quantitative RT-PCR verified that a glucose diet and *hyl-2* mutation decreased the gene expression of *cyp-25A1* and *ugt-63* genes (Figure S5). Notably, it is not the decreased expression of *cyp-25A1* and *ugt-63* that contributes to anoxia sensitivity since the knockdown of these genes using RNAi actually enhanced anoxia survival. The *ugt-63* and *cyp-25A1* genes, part of gene Cluster 4 and 5 (Figure 4, B and E), code for proteins that show homology to detoxification system proteins (Ma *et al.* 2013; Keller *et al.* 2014; Gems and McElwee 2005). The RNA-Seq data indicate that several *ugt* and *cyp* gene family members have a differential gene expression profile in the anoxia sensitive animals relative to controls (Figure 4A and Table S6). The *ugt* and *cyp* genes (*ugt-15*, *ugt-18*, *ugt-19*, *ugt-41*, *ugt-63*, *cyp-13A12*, *cyp-25A1*, and *cyp-33C8*), which are differentially expressed in N2 animals fed glucose and in *hyl-2(tm2031)* animals, were knocked-down via RNAi and the anoxia phenotype was examined. In all cases, relative to controls fed HT115 *E. coli*, the knockdown of the *ugt* genes and *cyp* genes increased the survival of 2 d of anoxia exposure (Figure 5, A and B). However, only *cyp-25A1(RNAi)* and *cyp-33C8(RNAi)* suppressed the sensitivity to 2 d of anoxia in the *hyl-2(tm2031)* animals (Figure 5, C and D). These data indicate that knockdown of some *ugt* and *cyp* genes enhances anoxia survival in animals fed a standard *E. coli* diet (no glucose supplement). We next examined if RNAi knockdown of *ugt-63* or *cyp-33C8* suppressed anoxia sensitivity in animals fed a glucose diet. The *ugt-63(RNAi)* or *cyp-33C8(RNAi)* animals fed a glucose diet did not survive anoxia exposure significantly differently than the HT115 control animals (Figure 6A). Note that the *E. coli* strain used here was HT115 and not OP50; this may account for a different survival rate in the N2 animals fed a 0.125% glucose-supplemented diet (Figure 6A and Figure 1C). In our analysis of the wild-type adult animals fed glucose we noted that the embryos they produced did not survive anoxia. The embryos of N2 hermaphrodites fed a 0.25% glucose-supplemented diet were very sensitive to anoxia, indicating that diet can impact the next generation’s capacity to respond to stress (Figure 6B). However, knockdown of *ugt-63* and *cyp-33C8* suppressed the anoxia sensitivity in the embryos from hermaphrodites fed a glucose diet (Figure 6B). Together, these data indicate that a glucose diet in the P0 generation reduces anoxia survival in the F1 generation embryo and that the knockdown of genes predicted to be a part of the xenobiotic and endobiotic phase I and II detoxification system suppresses this anoxia sensitivity.

## Discussion

Studies show that hyperglycemia worsens the outcome of an ischemic event such as stroke (Masrur *et al.* 2015; Sprafka *et al.* 1994; Megherbi *et al.* 2003; Bruno *et al.* 1999; Weir *et al.* 1997). However, it is not clear if specific physiological or molecular factors associated with type 2 diabetes impede oxygen deprivation responses. Using *C. elegans* we observed that a glucose-supplemented diet or altered ceramide metabolism (*hyl-2* mutant) decreases oxygen deprivation survival, and when these factors are combined there is a further decrease in anoxia survival. We used RNA-Seq to examine the transcriptomes of *hyl-2(tm2031)* animals fed a standard OP50 and OP50 glucose-supplemented diet and found that both the *hyl-2* mutation and glucose diet induces gene expression changes. The impact a glucose-supplemented diet has on the transcriptome of the wild-type animal is overlapping but not identical to that in the *hyl-2(tm2031)* animal (Garcia *et al.* 2015). Our approach to identify molecular changes that impact the oxygen deprivation response was based on comparative transcriptomics of anoxia sensitive animals (N2 glucose-fed and *hyl-2* mutant). This comparison identified 199 common transcripts that are differentially regulated in anoxia sensitive animals. Bioinformatics analysis of these transcripts shed a light on their functional significance revealing, in particular, the roles of their products in cuticle/collagen and ECM formation, innate immunity, and the xenobiotic and endobiotic phase I and II detoxification system.

A large proportion of the 199 transcripts that are differentially regulated in the anoxia sensitive animals represent the known or predicted collagen genes involved with the structural constituent of the cuticle and/or ECM (Figure 3 and Table S7). The *C. elegans* skin, composed of an epidermal epithelium and cuticle, provides a barrier for the animal and its environment (Page and Johnstone 2007; Chisholm and Hsiao 2012). Collagens make up a large portion of the protein component of the cuticle (Chisholm and Xu 2012) and the epidermis is also an organ that stores lipids (O’Rourke *et al.* 2009; Chisholm and Xu 2012). Collagen genes are typically expressed in particular larval stages or in a cyclic manner during cuticle synthesis for each molt (Johnstone 2000). It is not clear why many collagen genes are differentially regulated in animals fed a glucose diet or with altered ceramide biosynthesis. However, these findings are of interest in the context of complications associated with type 2 diabetes. Patients with diabetic nephropathy display changes to the ECM and an increase in collagen within the kidney (Kolset *et al.* 2012). Additionally, the skin problems associated with diabetics could result from several cellular changes including fragmentation of connective tissue and collagen, an increase in matrix metalloproteinases responsible for the fragmentation, and glycosylation of ECM proteins (Demirseren *et al.* 2014; Argyropoulos *et al.* 2016). Furthermore, in diabetic retinopathy, the ECM undergoes many structural changes including increases in collagens (Roy *et al.* 2015). *C. elegans* can serve as a model system to study how hyperglycemia or altered ceramide levels impact the structure and function of the ECM.

Interestingly, genes involved in innate immunity were also differentially regulated in the anoxia sensitive animals. Many of the genes that are involved with innate immunity in *C. elegans* have been identified by the study of the bacterial food and intestinal pathogens (*e.g.*, *Pseudomonas aeruginosa* and *Salmonella typhimurium*) (Gravato-Nobre and Hodgkin 2005; Ewbank and Pujol 2016). The standard *E. coli* strains (OP50 and HT115) used in our studies are not pathogenic *per se* to *C. elegans*; however, it is possible that if *C. elegans* is fed these bacteria along with glucose the animal might initiate an antimicrobial response. Some of the innate immunity genes are also differentially expressed in the *hyl-2(tm2031)* animals fed the standard *E. coli* diet. Previous studies have reported that the disruption of mitochondrial activity induces the pathogen-response and endobiotic and xenobiotic detoxification pathways (Liu *et al.* 2014; Melo and Ruvkun 2012; Govindan *et al.* 2015). Furthermore, the animals with altered regulation of ceramide biosynthesis pathways were deficient in responding to mitochondrial dysfunction (Liu *et al.* 2014). Thus, it is possible that an altered ceramide metabolism or a glucose diet disrupts mitochondrial functions leading to the expression of innate immunity genes. Another explanation for the expression of innate immunity genes in the anoxia sensitive animals is related to changes in cuticle structure; genetic mutations leading to abnormal cuticle development can trigger innate immune responses (Taffoni and Pujol 2015). Thus, it is possible that changes in cuticle components may be triggering downstream effects on genes involved with innate immunity. Further experiments would be needed to examine if these two processes are independently induced by a glucose diet or altered ceramide species or if they are mechanistically linked.

A recent study demonstrated that a glucose diet causes decreased membrane fluidity in worms with altered fatty acid metabolism (*paqr-2* and *iglr-2* mutants) (Svensk *et al.* 2016). PAQR-2, a homolog to the human AdipoR1/AdipoR2 proteins that regulate fatty acid metabolism, appears to work with IGLR-2 to maintain membrane homeostasis. The *iglr-2* transcript is upregulated in the *hyl-2(tm2031)* animals fed a glucose diet but not in wild-type animals fed the same diet. It is evident from the study by Svensk *et al.* (2016) that both *paqr-2* and *iglr-2* protect against glucose toxicity; perhaps the regulation of these genes in response to glucose is not at the transcriptional level. We found that a number of lipid metabolism genes are differentially expressed in the *hyl-2(tm2031)* mutant and the glucose-fed animals (Garcia *et al.* 2015). The GO terms—membrane lipid metabolism, sphingolipid metabolic process and cellular lipid metabolic process—were overrepresented in the *hyl-2(tm2031)*, *hyl-2(tm2031)* fed glucose, and N2 fed glucose animals. Ceramides are known to localize to mitochondria in *C. elegans* (Liu *et al.* 2014). Also, since there is an increase in lipids in glucose-fed animals, a glucose diet likely impacts the lipidomic profile (Garcia *et al.* 2015). Thus, it is possible that membrane fluidity of cells and perhaps organelles such as the mitochondria or ER are altered in glucose-fed animals and *hyl-2(tm2031)* mutants. Additional experiments could determine if this is the case and if this results in a compromised ability to survive anoxia exposure.

We hypothesized that the gene expression changes observed in an animal with altered ceramide metabolism or fed a glucose diet could negatively impact anoxia survival. We used RNAi to screen a subset of these genes and determined that the knockdown of the genes encoding the xenobiotic and endobiotic phase I and II detoxification system increased anoxia survival. The detoxification system is involved in the conversion of exogenous and endogenous molecules into hydrophilic products for excretion. The phase I detoxification system involves cytochrome P450s, which are largely responsible for the addition of oxygen to substrate molecules. The phase II detoxification system encompasses a broad range of enzymes including UDP-glycosyltransferases (UGTs), sulfotransferases, glutathione S-transferases, and N-acetyltransferases. The UGT enzymes reside in the ER membrane and catalyze the addition of the sugar glucuronic acid to lipophilic endobiotics and xenobiotics. In *C. elegans*, these detoxification gene families are large but very few functional analysis studies have been conducted. Interestingly, a member of the *cyp* gene family (*cyp-13A12*) was implicated in the reoxygenation response in *C. elegans* (Ma *et al.* 2013). Furthermore, there is evidence that *C. elegans* have a surveillance system that monitors core cellular activities (*e.g.*, translation, respiration, and secretory pathways) and engage in behavioral, immune, and detoxification responses when these core cellular activities are compromised (Melo and Ruvkun 2012). The *cyp-35B1*::*GFP* reporter, expressed in the intestine, is induced by the inactivation of genes involved in protein synthesis, mitochondrial function, metabolism, vascular trafficking, and cuticle specific functions, suggesting that there is some signaling between different organ types (Melo and Ruvkun 2012). In our study, most of the detoxification genes differentially regulated in the glucose-fed and *hyl-2(tm2031)* animals were upregulated.

It is not yet clear why knockdown of the detoxification genes via RNAi increases anoxia survival. It is possible that core cellular processes are disrupted by a glucose diet or altered ceramide metabolism and this, in turn, induces expressional changes in the detoxification genes, which ultimately leads to an increased flux of the detoxification pathway. Perhaps, an increased flux of the detoxification pathway compromises anoxia survival by utilizing molecules (such as stored oxygen) needed to survive anoxia. In humans, UGTs promote the excretion of toxic metabolites, including pharmaceuticals, in bile or urine and facilitate the homeostasis of endogenous molecules such as bilirubin (end-product of heme metabolism), some steroid hormones, and fatty acids (Guillemette 2003). Bilirubin, in the past thought of only as a toxic waste product, may also possess potent antioxidant capabilities at physiologically relevant oxygen concentrations. Though a sustained high concentration of bilirubin is neurotoxic, low to moderately increased levels have been shown to be cytoprotective (Stocker *et al.* 1987; Hegyi *et al.* 1994; Erdogan *et al.* 2012). This supports the idea that the detoxification system can modulate the stress response through secondary mechanisms, and that it is not necessarily paradoxical that knockdown of some detoxification genes may be beneficial in some circumstances. Thus, it is possible that knockdown of the detoxification genes in *C. elegans* alters the level of a target molecule which, in turn, alters oxygen deprivation responses. However, since the detoxification genes are understudied in *C. elegans*, their substrates and/or targets are currently unclear.

We found that P0 animals fed a glucose diet led to a reduced anoxia survival in the F1 generation indicating that the parental diet negatively impacted anoxia resistance in offspring. Knockdown of *ugt-63* or *cyp-33C8* in the P0 generation fed a glucose diet suppressed anoxia sensitivity observed in the F1 embryo. However, the mechanism involved with this transgenerational preconditioning effect is not understood. Others have analyzed the impact that a high glucose diet has on subsequent generations (Tauffenberger and Parker 2014). They found that a 2% glucose diet fed to the P0 generation reduced progeny production but increased resistance to the oxidative stress inducer juglone in the F1 generation. We have not observed glucose-induced stress resistance so perhaps differences in methodology (*e.g.*, glucose concentration or specific type of stress) impacts how the animal responds to the stress. Future studies are needed to understand the functional role the detoxification genes have in oxygen deprivation survival, particularly relative to altered ceramide metabolism and elevated glucose level, in both the parental and subsequent generations.

## 

## Supplementary Material

Supplemental Material
