# Supplementary material for: Glucose or Altered Ceramide Biosynthesis Mediate Oxygen Deprivation Sensitivity Through Novel Pathways Revealed by Transcriptome Analysis in Caenorhabditis elegans
Source: G3 (Bethesda). 2016 Aug 5;6(10):3149–60. doi: 10.1534/g3.116.031583 (PMC5068937; doi:10.1534/g3.116.031583)
Supplement: Supplemental Material [file supp_6_10_3149__index.html]

Glucose or Altered Ceramide Biosynthesis Mediate Oxygen Deprivation Sensitivity Through Novel Pathways Revealed by Transcriptome Analysis in Caenorhabditis elegans — Supplemental Material 

# Glucose or Altered Ceramide Biosynthesis Mediate Oxygen Deprivation Sensitivity Through Novel Pathways Revealed by Transcriptome Analysis in *Caenorhabditis elegans*

## Supplemental Material for Ladage *et al.*, 2016

**Files in this Data Supplement:**

- Figure S1 - RNA-Seq analysis reveals differentially regulated genes in *hyl-2(tm2031)* animals. (.jpg, 109 KB)
- Figure S2 - Transcripts were categorized based on Gene Ontology (GO) annotations for biological functions; the number of genes for each category is displayed in the pie-chart. (.jpg, 737 KB)
- Figure S3 - A heatmap showing the transcriptional profile for genes differentially expressed in N2 animals fed a glucose diet and in *hyl-2(tm2031)* mutants. (.jpg, 488 KB)
- Figure S4 - Relative to N2 control, RNAi of the indicated genes did not significantly increase anoxia survival. (.jpg, 53 KB)
- Figure S5 - Validation of RNA-Seq analysis by qRT-PCR for *cyp-25A1* and *ugt-63* transcripts that showed downregulation in animals fed a glucose diet and the *hyl-2(tm2031)* mutant relative to wild-type N2 animals fed a standard OP50 diet. (.jpg, 76 KB)
- Table S1 - Upregulated transcripts in *hyl-2* animals. (.xlsx, 177 KB)
- Table S2 - Upregulated transcripts in *hyl-2* animals fed a glucose diet. (.xlsx, 797 KB)
- Table S3 - Transcripts upregulated in N2 and *hyl-2* animals fed a glucose diet. (.xlsx, 209 KB)
- Table S4 - Transcripts downregulated in N2 and *hyl-2* animals fed a glucose diet. (.xlsx, 160 KB)
- Table S5 - Lipid metabolism gene datasets. (.xlsx, 41 KB)
- Table S6 - Common upregulated transcripts in anoxia sensitive animals (N2 glucose and *hyl-2(tm2031)*). (.xls, 425 KB)
- Table S7 - Babelomics analysis of the 199 common transcripts. (.xlsx, 30 KB)
- Table S8 - Cluster 1 transcripts. (.xlsx, 67 KB)
