# Supplementary figures and images for: Glucose or Altered Ceramide Biosynthesis Mediate Oxygen Deprivation Sensitivity Through Novel Pathways Revealed by Transcriptome Analysis in Caenorhabditis elegans
Source: G3 (Bethesda). 2016 Aug 5;6(10):3149–60. doi: 10.1534/g3.116.031583 (PMC5068937; doi:10.1534/g3.116.031583)

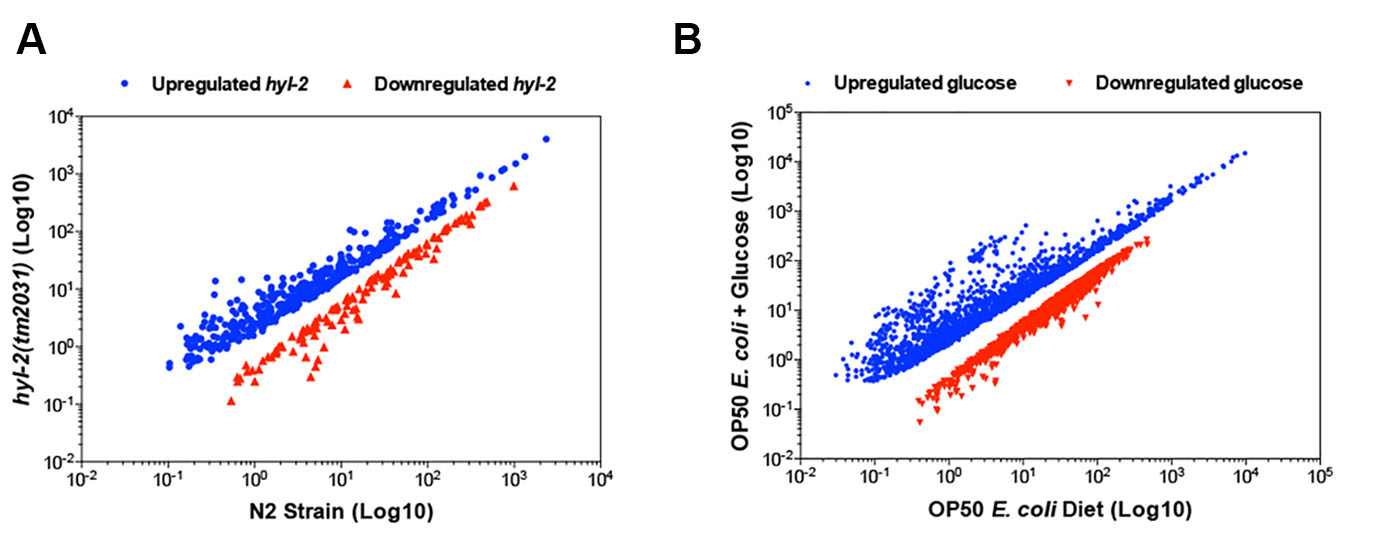

Supplement: Supplemental Material [file supp_g3.116.031583_FigureS1.jpg]

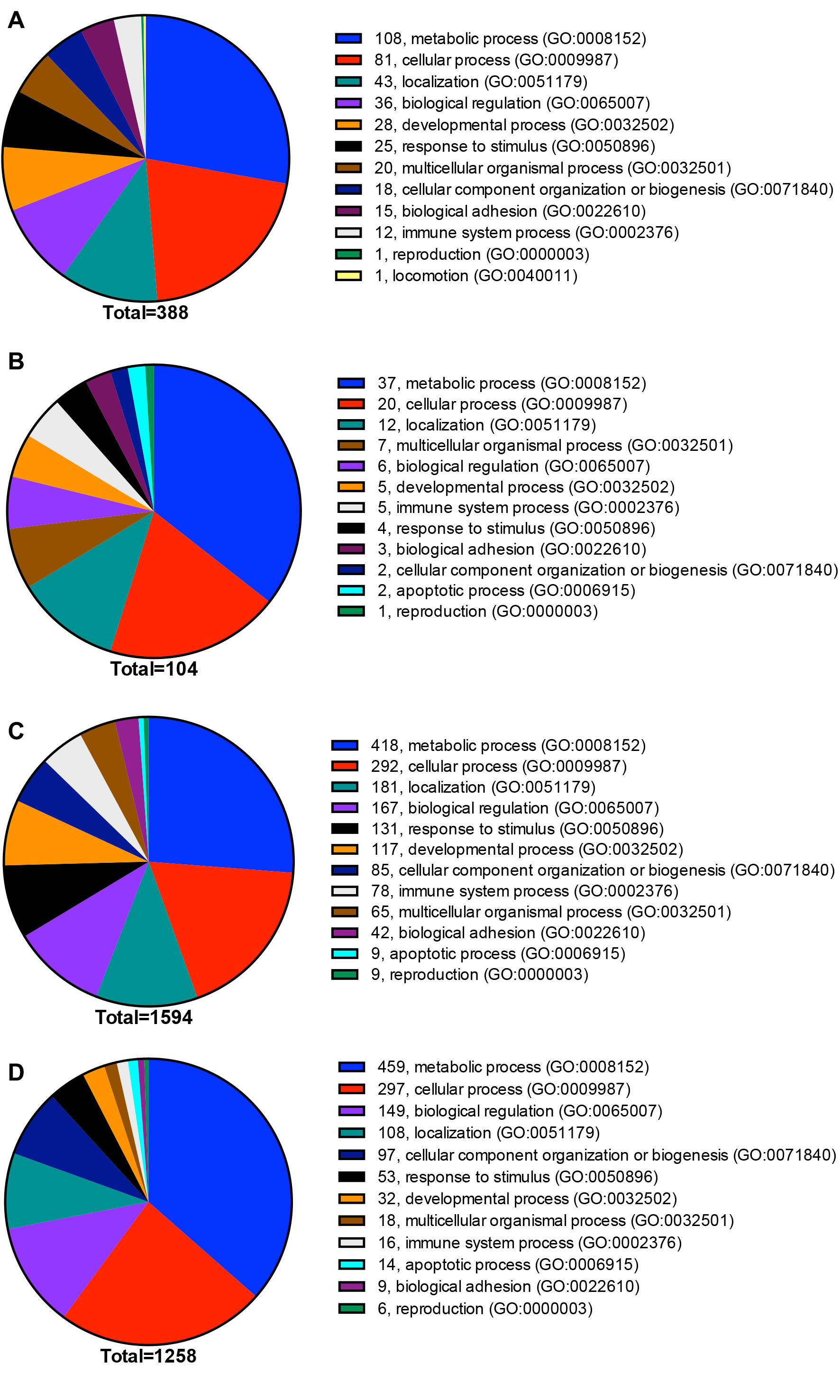

Supplement: Supplemental Material [file supp_g3.116.031583_FigureS2.jpg]

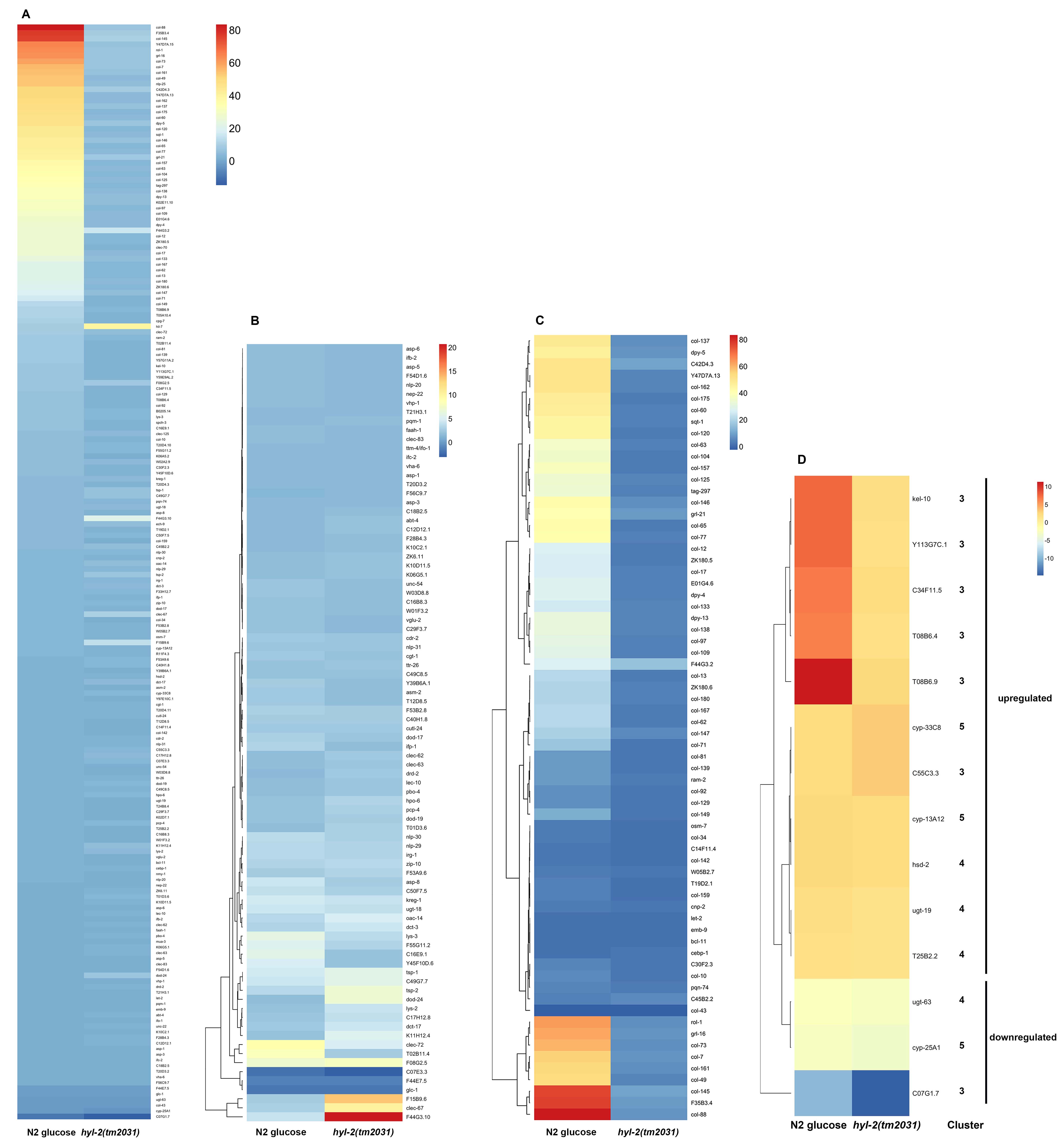

Supplement: Supplemental Material [file supp_g3.116.031583_FigureS3.jpg]

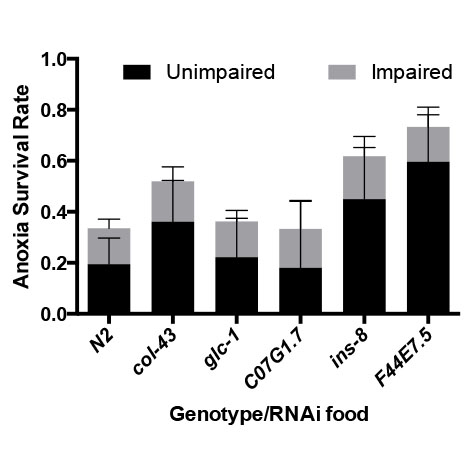

Supplement: Supplemental Material [file supp_g3.116.031583_FigureS4.jpg]

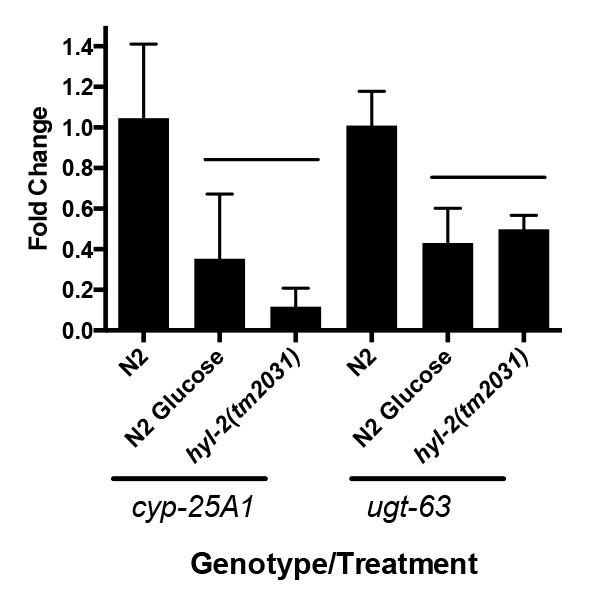

Supplement: Supplemental Material [file supp_g3.116.031583_FigureS5.jpg]
